# A pan-cancer analysis identifies SOAT1 as an immunological and prognostic biomarker

**DOI:** 10.32604/or.2023.027112

**Published:** 2023-04-10

**Authors:** YANGQING HUANG, XINLAN ZHOU, XIUFEN LI, DAN HUANG, ZHONG FANG, RONGRONG DING

**Affiliations:** 1 Department of Hepatobiliary Medicine, Shanghai Public Health Clinical Center, Fudan University, Shanghai, China; 2 Department of Hepatobiliary Surgery, Shanghai Public Health Clinical Center, Fudan University, Shanghai, China; 3 Liver Cancer Institute of Zhongshan Hospital and Key Laboratory of Carcinogenesis and Cancer Invasion (Ministry of Education), School of Basic Medical Sciences, Fudan University, Shanghai, China

**Keywords:** SOAT1, Pan-cancer, Immune infiltration, Prognostic biomarker, Tumor microenvironment

## Abstract

Sterol o-acyltransferase1 (SOAT1) is an enzyme that regulates lipid metabolism. Nevertheless, the predictive value of SOAT1 regarding immune responses in cancer is not fully understood. Herein, we aimed to expound the predictive value and the potential biological functions of SOAT1 in pan-cancer. Raw data related to SOAT1 expression in 33 different types of cancer were acquired from The Cancer Genome Atlas (TCGA) and Genotype-Tissue Expression (GTEx) databases. SOAT1 expression was significantly increased in most cancers and showed a distinct correlation with prognosis. This enhanced expression of the SOAT1 gene was confirmed by evaluating SOAT1 protein expression using tissue microarrays. In addition, we found significant positive associations between SOAT1 expression levels and infiltrating immune cells, including T cells, neutrophils, and macrophages. Moreover, the co-expression analysis between SOAT1 and immune genes showed that many immune-related genes were increased with enhanced SOAT1 expression. A gene set enrichment analysis (GSEA) revealed that the expression of SOAT1 correlated with the tumor microenvironment, adaptive immune response, interferon signaling, and cytokine signaling. These findings indicate that SOAT1 is a potential candidate marker for predicting prognosis and a promising target for tumor immunotherapy in cancers.

## Introduction

Cancer is the leading cause of death and poor quality of life worldwide [1]. To date, the treatment of cancers remains a substantial challenge in the medical field. Cancer is a complex disease involving interactions between tumor cells and immune responses. Interactions between tumors and immune cells have become a research focus, and immunotherapies targeting immune checkpoints or other strategies have been developed to treat cancers [2]. Immunotherapy demonstrates efficacy in various cancer types by reactivating the adaptive and innate immune system and is considered a substitute for traditional cancer therapy [3]. However, it remains necessary to clarify the interaction between cancers and immune responses to find new targets for developing novel immunotherapies. The deep development and improvement of public resources, such as the Genotype-Tissue Expression (GTEx) and Cancer Genome Atlas (TCGA) databases, has enabled the identification of new immunotherapy candidates via pan-cancer gene analysis and the assessment of associations of these genes with clinical outcomes or the related immune status [4].

Several studies have revealed an important relationship between lipid metabolism and the occurrence or development of malignant tumors [5,6]. Sterol o-acyltransferase (SOAT) is a crucial lipid metabolism enzyme that can esterify cholesterol into cholesterol esters in adipocytes [7,8]. SOAT has two isomers, SOAT1 and SOAT2. SOAT1 is a common histocyte subtype that mainly exists in the endoplasmic reticulum [9]. SOAT1 has also been found to be associated with the progression of atherosclerosis, Alzheimer’s disease, and several cancers [10–14]. However, the biological function of SOAT1 in regulating tumor progression has not been elucidated.

Previous studies on the role of SOAT1 in tumors were restricted to specific types of cancer. Here, we conducted a pan-cancer study to evaluate SOAT1 levels and their relationship with prognosis using TCGA, GTEx, and cBioPortal databases. We then analyzed the potential associations between the expression of SOAT1 and tumor-infiltrating immune cells or related immune markers. These findings implied that SOAT1 potentially influences cancer prognosis partly through an important role in tumor immunity. Thus, SOAT1 is a potential marker for predicting prognosis and a promising tumor immunotherapy target.

## Methods

### Determining the diversity of SOAT1 expression in different human cancers

Gene expression levels in 33 different cancers were obtained from the GTEx database (https://commonfund.nih.gov/GTEx). RNA sequence, mutation, clinicopathological, and survival data were collected from the TCGA database (9784 samples from 33 types of tumors) using UCSC Xena (https://xena.ucsc.edu/).

SOAT1 expression data were Log2 transformed, and two sets of t-tests were conducted for all tumor types. *p* < 0.05 was considered to indicate a significant difference between tumor and normal tissues. Data analyses were performed using R software (Version 4.0.4; https://www.R-project.org), and the R package “ggpubr” was used to generate box plots.

### Detection of SOAT1 protein in cancer and paracancerous tissue microarrays

We next identified SOAT protein levels in different types of cancer. SOAT1 expression was assessed through immunohistochemistry via a multiple-cancer tissue microarray (HOrgC120PG05, Shanghai Outdo Biotech Co., Ltd., Shanghai, China). Rabbit anti-SOAT1 polyclonal antibody was obtained from Ab-Mart (Shanghai, China) and used at 1:100 dilution. A total of 120 paraffin-embedded organic tissues were obtained for analysis, including 9 normal tissue samples and samples from 10 types of cancer and their paired paracancerous tissue. Eight breast cancer tissues were also obtained but lacked paracancerous or normal tissue controls; thus, these data were excluded.

### Association of SOAT1 expression with DNA methylation

Methylation, a form of DNA modification, usually regulates gene transcription and can potentially cause tumorigenesis [15]. Therefore, we downloaded methylation data from cBioPortal (www.cbioportal.org) and then correlated SOAT1 levels with promoter methylation in each tumor. The association between SOAT1 methylation and prognosis was determined via a Kaplan–Meier survival analysis, including overall survival (OS) and disease-specific survival (DSS) rates and the progression-free and disease-free intervals (PFI and DFI, respectively).

### Association between SOAT1 and TMB or MSI in pan-cancer

Tumor Mutation Burden (TMB) is a quantitative immune biomarker reflecting the number of mutations in cancer cells [16]. Tumor Microsatellite Instability (MSI), generated from DNA mismatch repair, has been reported to be associated with patient prognosis [17]. TMB was evaluated with the Perl script after dividing by the full length of exons (number of mutated events per 1 million bases) to study the number of mutations in each tumor. The MSI score was further determined from TCGA data. Correlations between TMB and MSI with cancer gene expression were investigated via the “cor.test” command of Spearman’s method. Both indicators are shown in a lollipop chart designed by the R-package “ggdotchart”.

### Immune cell infiltration in pan-cancer

We downloaded and calculated the immune cell infiltration levels in the TCGA dataset using the ImmuCellAI database (http://bioinfo.life.hust.edu.cn/web/ImmuCellAI/). Patients were divided into two groups per TCGA tumor type (high and low SOAT1 mRNA level according to the median gene expression level) to compare the extent of immune cell infiltration.

Moreover, we conducted a co-expression analysis of SOAT1 and immune-related genes, such as immune activation, immunosuppression, chemokine, major histocompatibility complex (MHC), and chemokine receptors. R-package “limma”, the “reshape2”, and “RColorBreyer” packages were used to present the results.

### Gene set enrichment analysis

A gene set enrichment analysis (GSEA) was used to investigate biological signaling pathways. Gene ontology (GO) and Reactome gene sets were selected for GSEA. Enrichment analysis was performed using the R package “clusterProfiler”.

### Prognostic analyses of SOAT1 expression in pan-cancers

Survival information for each sample was downloaded from the TCGA dataset, and the correlation between SOAT1 levels and clinical outcomes was studied. Four indicators, OS, DSS, DFI, and PFI outcomes, were further evaluated. The Kaplan–Meier method and log-rank test were then employed for the survival analysis (*p* < 0.05). Survival curves were generated using the R packages “survival” and “survminer”. Moreover, Cox analysis was performed using the R packages “survival” and “forestplot” to determine the association between SOAT1 level and survival.

### Statistical analysis

Gene expression data from the TCGA and GTEx databases were evaluated by Student’s *t*-test. Kaplan–Meier curves, log-rank tests, and Cox proportional hazard regression models were employed to analyze patient survival data. Associations between SOAT1 levels and the abundance scores of immune cells were evaluated by Spearman’s correlation. All statistical analyses were performed using R packages. *p* < 0.05 was considered to indicate significance.

## Results

### SOAT1 gene expression in pan-cancer

**Figure 1:**
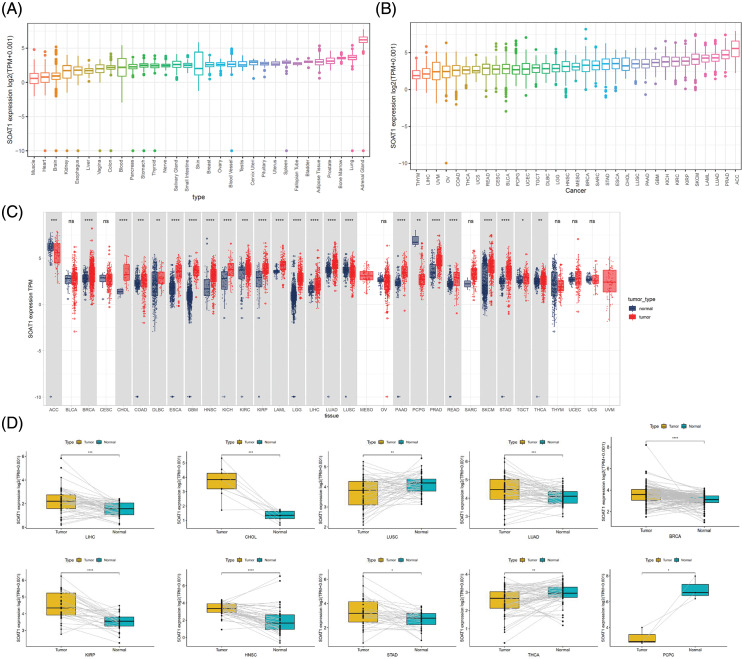
Distinct expression of SOAT1. (A) SOAT1 expression in normal tissues. (B) SOAT1 expression in tumors. (C) Comparison of the levels of SOAT1 expression between cancer and normal samples. (D) Comparison of the levels of SOAT1 in paired tumor and normal tissues. *p < 0.05, **p < 0.01, ***p < 0.001, ****p < 0.0001.

SOAT1 gene expression in tissues was explored using the GTEx data set (Fig. 1A). Expression of SOAT1 was elevated in normal bladder, adipose, lung, and adrenal gland tissue, whereas most other normal tissues demonstrated low SOAT1 expression. We then analyzed SOAT1 expression in different cancers. The values are shown in Fig. 1B from lowest to highest. The highest level of SOAT1 expression was observed in adrenocortical carcinoma (ACC), and the lowest in thymoma (THYM).

Using TCGA data, SOAT1 expression levels were compared between tumor and paired normal tissues. Since normal sample data were lacking for mesothelioma (MESO) and uveal melanoma (UVM), significant differences in SOAT1 expression between tumor and normal tissues were only compared in 24 cancers. Among these cancers, SOAT1 was highly expressed in invasive breast carcinoma (BRCA), cholangiocarcinoma (CHOL), colon adenocarcinoma (COAD), lymphoid neoplasm diffuse large B-cell lymphoma (DLBC), esophageal carcinoma (ESCA), glioblastoma multiforme (GBM), head and neck squamous cell (HNSC), kidney chromophobe (KICH), kidney renal clear cell carcinoma (KIRC), kidney renal papillary cell carcinoma (KIRP), testicular germ cell tumors (TGCT), acute myeloid leukemia (LAML), brain lower grade glioma (LGG), pancreatic adenocarcinoma (PAAD), liver hepatocellular carcinoma (LIHC), lung adenocarcinoma (LUAD), prostate adenocarcinoma (PRAD), rectum adenocarcinoma (READ), skin cutaneous melanoma (SKCM), stomach adenocarcinoma (STAD), and thyroid carcinoma (THCA). On the contrary, SOAT1 expression was low in adrenocortical carcinoma (ACC), lung squamous cell carcinoma (LUSC), and pheochromocytoma and paraganglioma (PCPG). There was no significant difference in SOAT1 expression in seven cancers, bladder urothelial carcinoma (BLCA), cervical squamous cell carcinoma and endocervical adenocarcinoma (CESC), ovarian serous cystadenocarcinoma (OV), sarcoma (SARC), THYM, uterine *corpus* endometrial carcinoma (UCEC), and uterine carcinosarcoma (UCS) (Fig. 1C). Furthermore, similar expression differences in these cancers were observed in matched tumor and normal tissues (Fig. 1D).

**SUPPLEMENTARY FIGURE S1:**
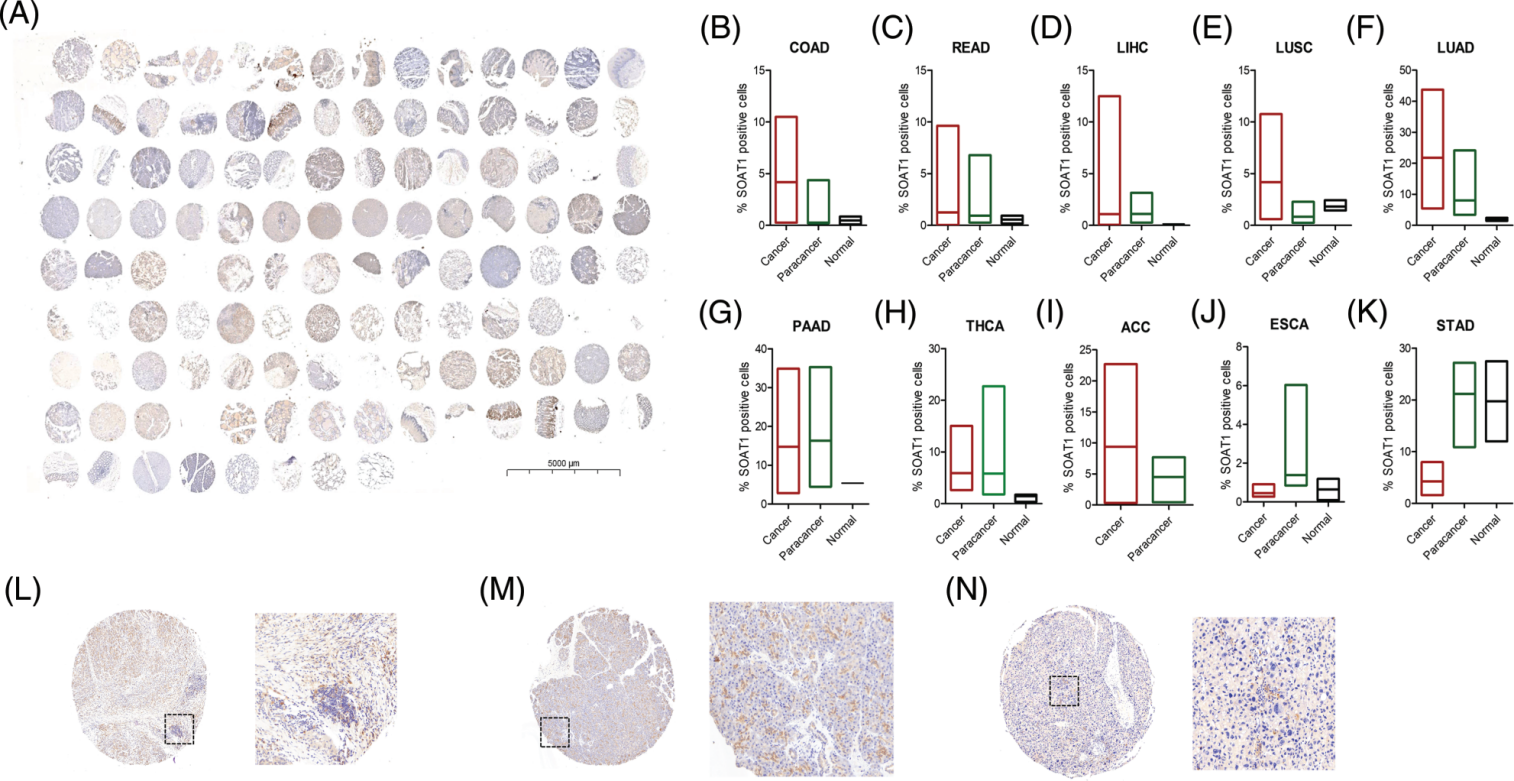
Detection of SOAT1 protein in tissue microarrays. (A) Tissue microarray, containing 10 different cancers and their paracancerous samples, was stained with anti-SOAT1 antibody. Percentage of SOAT1 positive cells were calculated and compared in each cancer and their paracancerous samples (B–K). (L) Staining SOAT1 in cancer tissue from PAAD. (M) Staining SOAT1 in paracancerous tissue from PAAD. (N) Staining SOAT1 in cancer tissue from HCC.

After analyzing SOAT1 gene expression in pan-cancer databases, we next evaluated SOAT1 protein levels. Therefore, we employed tissue microarrays covering 10 different cancers and their paracancerous tissues for SOAT1 IHC staining (Suppl. Fig. S1A). Consistent with our gene expression data, in most types of cancer, SOAT1 is higher in the cancer and paracancerous tissues than in the paired normal tissues (Suppl. Figs. S1B–S1H). However, a strong non-specific signal was observed in paracancerous and normal stomach tissues (Suppl. Fig. S1K). SOAT1 levels were higher in COAD, READ, LIHC, LUSC, LUAD, and ACC samples than in their associated paracancerous samples (Suppl. Figs. S1B–S1F). Interestingly, the percentage of positive cells in PAAD and paracancerous tissues was almost equal (Suppl. Fig. S1G); however, the expression location was substantially altered. SOAT1 was detected in the cytoplasm of PAAD cells (Suppl. Fig. S1L), whereas it was found in the nucleus of paracancerous tissue (Suppl. Fig. S1M). In addition, in THCA and ESCA (Suppl. Figs. S1H and S1J), higher SOAT1 protein levels were observed in paracancerous tissues than in cancer tissues, suggesting SOAT1 expression is affected by the tumor microenvironment (TME) in addition to the cells themselves.

### Association of SOAT1 expression with Methylation, TMB, MSI, and TME

**SUPPLEMENTARY FIGURE S2:**
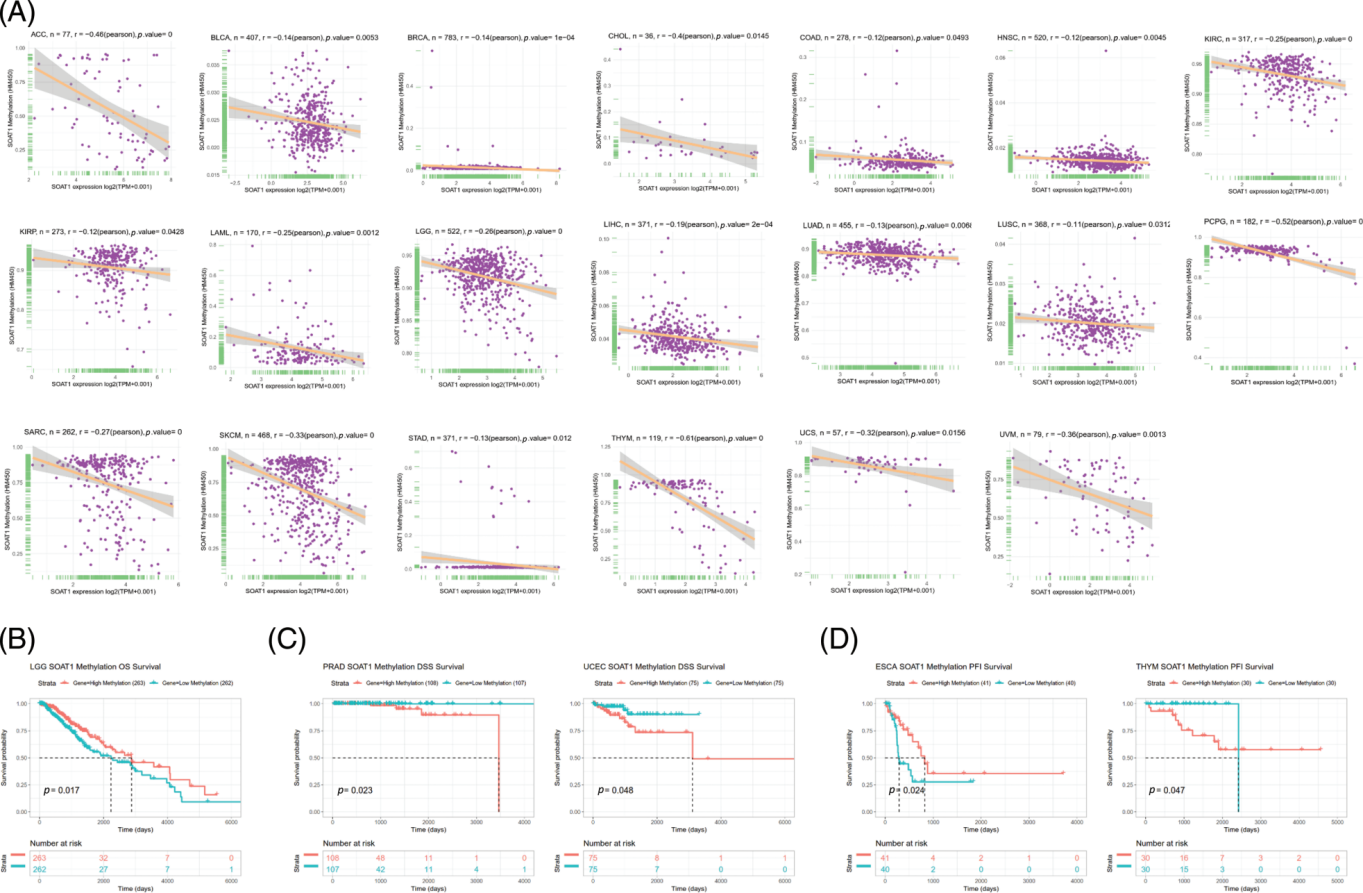
Correlation between SOAT1 expression and gene promoter methylation. (A) Correlation between SOAT1 expression and gene promoter methylation in vary cancers. (B) Correlation between SOAT1 methylation and OS. (C) Correlation between SOAT1 and DSS. (D) Correlation between SOAT1 and PFI.

Promoter methylation typically regulates gene expression; thus, we calculated the methylation level of the SOAT1 promoter using the cBioPortal dataset. Significant reverse correlations between SOAT1 expression and methylation levels in 20 tumors, ACC, BLCA, BRCA, CHOL, COAD, KIRC, KIRP, LAML, LGG, LIHC, LUAD, LUSC, PCPG, SARC, SKCM, STAD, HNSC, THYM, UCS, and UVM, were observed (Suppl. Fig. S2A). Then, Kaplan–Meier survival analyses were used to determine the diagnostic value of SOAT1 promoter methylation for patient prognosis. We found that SOAT1 methylation might be a protective factor in patients with LGG regarding OS outcomes (Suppl. Fig. S2B). With respect to DSS outcomes, SOAT1 methylation was a detrimental factor in patients with PRAD and UCEC (Suppl. Fig. S2C). Moreover, the analysis of PFI data revealed that SOAT1 methylation played a protective role in ESCA but a detrimental role in THYM (Suppl. Fig. S2D). These findings indicate that SOAT1 expression levels are altered by promoter methylation and that such methylation regulates its expression in pan-cancer progression.

**Figure 2:**
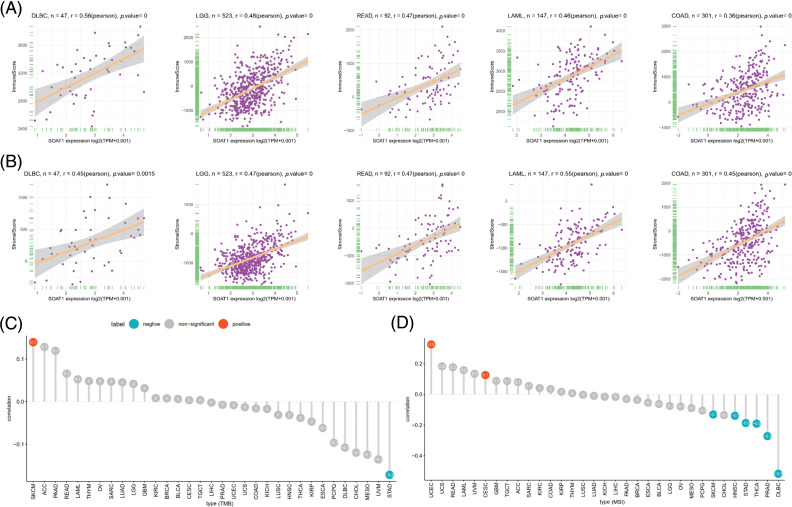
Association between SOAT1 expression and TMB and MSI. (A) Relationship between SOAT1 and immune scores in DLBC, LGG, READ, LAML, and COAD. (B) Relationship between SOAT1 and immune scores in DLBC, LGG, READ, LAML, and COAD. (C) Lollipop chart showing the relationship between SOAT1 and TMB. (D) Lollipop chart showing the relationship between SOAT1 and MSI. The five cancers with the highest correlation coefficients between SOAT1 and TMB.

The tumor microenvironment (TME) plays an important role in the heterogeneity between different cancer cells, thereby increasing multidrug resistance and leading to the progression and metastasis of cancer cells [18]. We also validated the prognostic value of SOAT1 in various cancers; hence, it is necessary to further study the relationship between TME and SOAT1 levels in different cancers. The Estimation of STromal and Immune cells in MAlignant Tumor tissues using Expression data (ESTIMATE) algorithm was then used to calculate the stromal and immune cell scores in pan-cancer, and the correlation between the scores and SOAT1 expression levels was analyzed. We found that SOAT1 levels positively correlated with immune (Fig. 2A) and stromal scores (Fig. 2B) in DLBC, LGG, READ, LAML, and COAD. These findings showed a crucial role of SOAT1 in MSI, TMB, and TME.

TMB is a critical biomarker for sensitivity to treatments involving immune checkpoint inhibitors, such as PD-1/PD-L1 [19,20]. MSI has been detected in ~15% of colorectal cancers and has also been reported as a biomarker for anti-PD-1 treatment [21,22]. Thus, investigating the relationships between SOAT1 expression level and TMB and MBI in different types of cancer is warranted. Our results revealed that SOAT1 levels positively correlate with TMB in SKCM, but negatively correlate in STAD. In addition, SOAT1 expression was significantly correlated with MSI in UCEC and CESC (Fig. 2C). Moreover, expression of SOAT1 was reversely correlated with MSI in SKCM, HNSC, STAD, THCA, PRAD, and DLBC (Fig. 2D).

### Correlations between SOAT1 levels and infiltrating immune cells

**Figure 3:**
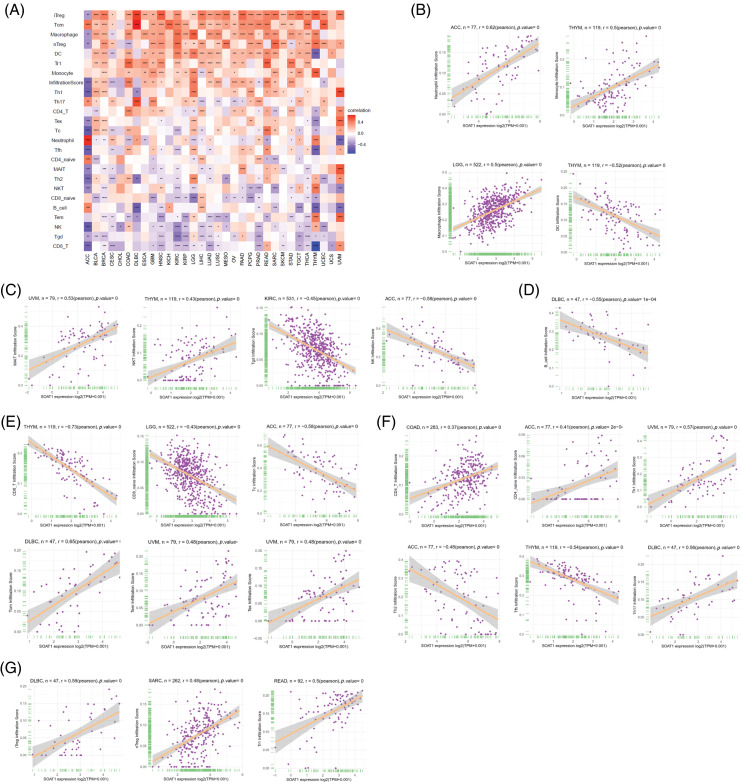
Relationships between infiltrating immune cells and SOAT1 expression. Correlations between infiltrating immune cells and SOAT1 in pan-cancer (A). Relations between SOAT1 and myeloid cells (B), innate and innate-like lymphocytes (C), B cells (D), CD8+ T cells (E), CD4+ T cells (F), and regulatory T cells (G).

We then correlated SOAT1 expression with infiltrating immune cells. Our results showed that the levels of immune cell infiltration were dramatically increased with enhanced SOAT1 expression in different types of tumors. SOAT1 expression was significantly associated with the infiltration of dendritic cells (DC), regulatory T cells (iTreg, nTreg, and Tr1), T follicular helpers (TFH), central memory T cells (Tcm), and macrophages in most tumors (Fig. 3A). By contrast, SOAT1 expression levels were reversely correlated with infiltration of CD8+ T cells, Effector memory T Cell (Tem), gamma delta T (Tgd), NKT, NK, and B cells in most tumors (Fig. 3A). Cancers with the most significant correlation between the types of infiltrating immune cells and SOAT1 expression levels were then analyzed. Briefly, myeloid cells, including neutrophils, monocytes, macrophages, and dendritic cells, presented the highest correlations with SOAT1 expression in ACC, THYM, LGG, and THYM, respectively (Fig. 3B). In lymphoid populations, SOAT1 levels were most correlated with innate and innate-like lymphocytes, including mucosal-associated invariant T cells (MAIT), NKT, γδT, and NK in UVM, THYM, KIRC, and ACC (Fig. 3C). Moreover, in DLBC, THYM, LGG, and ACC, SOAT1 expression demonstrated strong reverse correlations with the infiltration of B and CD8+ cell populations (Figs. 3D and 3E) but significant positive correlations with CD4+ and regulatory cell types (Figs. 3F and 3G). The pan-cancer data are shown in Suppl. Table S1. These infiltrations can also be simply observed in tissue microarrays, as shown in Suppl. Figs. S1L and S1N, indicating that SOAT1 protein is accompanied by an increase in infiltrating immune cells. These results suggest that SOAT1 might play an important role in regulating the tumor immune microenvironment.

### Associations between SOAT1 levels and immune checkpoint markers

**Figure 4:**
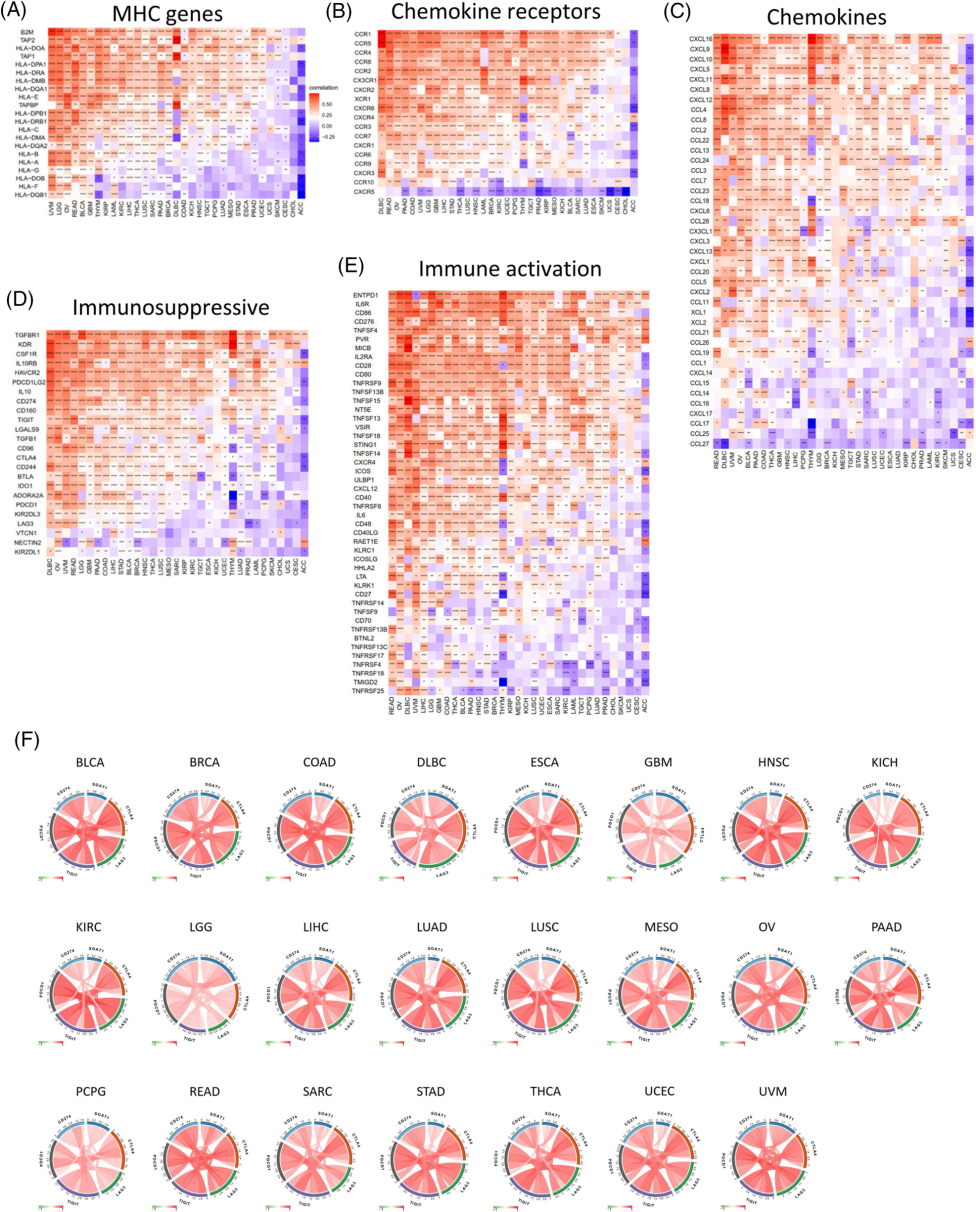
Co-expression of SOAT1 and immune-related genes. MHC genes (A), chemokines and chemokine receptors genes (B, C), and immune suppressive and activation genes (D, E). Correlation of SOAT1 levels with main immune checkpoint markers in various cancers (F).

A gene co-expression analysis was further used to study the associations between SOAT1 expression levels and immune-related genes in pan-cancer. Genes related to activation or immunosuppression of immune responses, chemokines, chemokine receptors, and MHC molecules were analyzed. The heat map showed that most immune-related genes, including MHCs (Fig. 4A), chemokines (Fig. 4B), chemokine receptors (Fig. 4C), and activation and suppression genes (Fig. 4D and 4E), were co-expressed with SOAT1. These positive correlations were observed in most tumors, except for CHOL and ACC.

We next investigated the correlations between SOAT1 expression and main immune checkpoint gene expression, including CD274, PDCD1, TIGIT, LAG3, and CTLA4. Interestingly, we found that in 23 tumors, including BLCA, BRAC, COAD, and DLBC, the level of SOAT1 was correlated with the five immune checkpoints (Fig. 4F). These results suggest SOAT1 might play a critical role in immune escape in these tumors and is a promising target in tumor immunotherapy.

### Functional enrichment analyses of SOAT1 in pan-cancer

**Figure 5:**
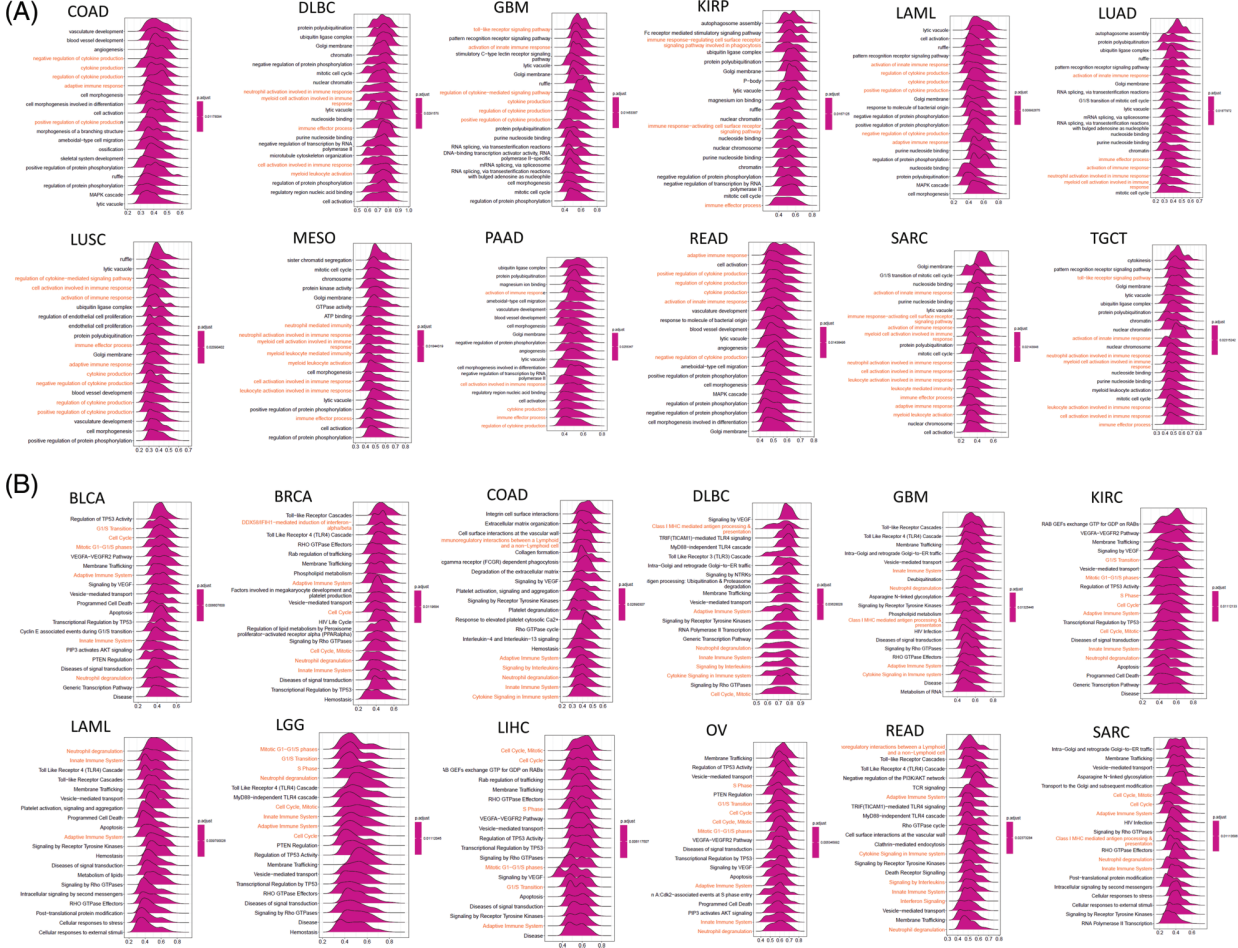
GSEA of SOAT1 in various cancers. (A) GO functional annotation of SOAT1 expression. (B) Reactome pathway analysis of SOAT1 expression

SOAT1-associated pathways were explored by GSEA in 33 types of tumors from the TCGA. SOAT1 was positively regulated and provided several immune-related functions. These activities included (i) cytokine production, (ii) adaptive immune response, (iii) cell activation involved in immune response, and (iv) activating cell surface receptor signaling pathways (Fig. 5A). Moreover, SOAT1 expression positively regulated several immune pathways such as (i) adaptive immune system, (ii) innate immune system, (iii) neutrophil degranulation, (iv) interferon signaling, (v) cytokine signaling in immune system, and (vi) interleukins signaling (Fig. 5B), suggesting that SOAT1 is involved in these immune pathways.

### Prognostic value of SOAT1 in pan-cancer

**Figure 6:**
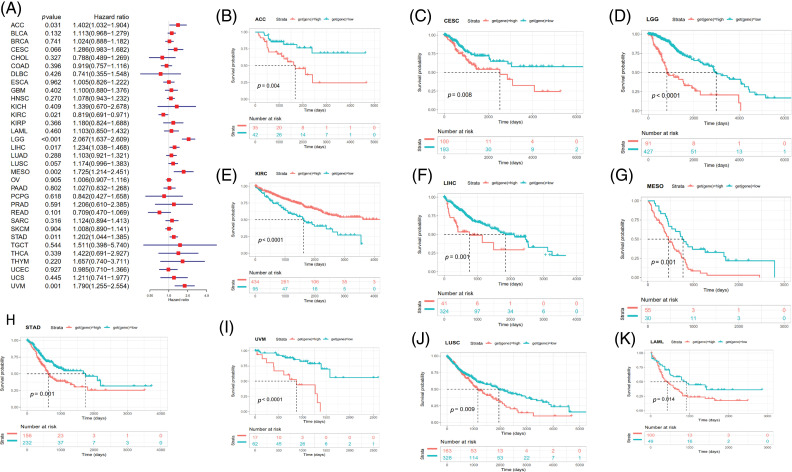
Relationship between SOAT1 levels and overall survival (OS) time in days. (A) Forest plots of OS associations in 33 types of tumors. (B–K) Kaplan–Meier analysis for the correlation between SOAT1 expression and OS outcomes.

Survival analyses for each cancer were performed to examine the association between SOAT1 expression and prognosis. Cox proportional hazards model analysis showed that SOAT1 levels were associated with OS outcomes in ACC (*p* = 0.031), KIRC (*p* = 0.021), LGG (*p* < 0.001), LIHC (*p* = 0.017), MESO (*p* = 0.002), STAD (*p* = 0.011), and UVM (*p* = 0.001) (Fig. 6A), suggesting SOAT1 was a high-risk gene in ACC, LGG, MESO, STAD, LIHC, and UVM, whereas in KIRC, it was low-risk.

Then, we conducted Kaplan–Meier survival analyses and found that in patients with ACC (*p* = 0.004), CESC (*p* = 0.008), LGG (*p* < 0.0001), LIHC (*p* = 0.001), MESO (*p* = 0.001), LUSC (*p* = 0.009), UVM (*p* < 0.0001), STAD (*p* = 0.001), and LAML (*p* = 0.014), enhanced SOAT1 expression was accompanied by poor OS outcomes (Figs. 6B–6D and 6F–6K); however, in patients with KIRC (*p* < 0.0001), SOAT1 was a low-risk gene (Fig. 6E).

**SUPPLEMENTARY FIGURE S3:**
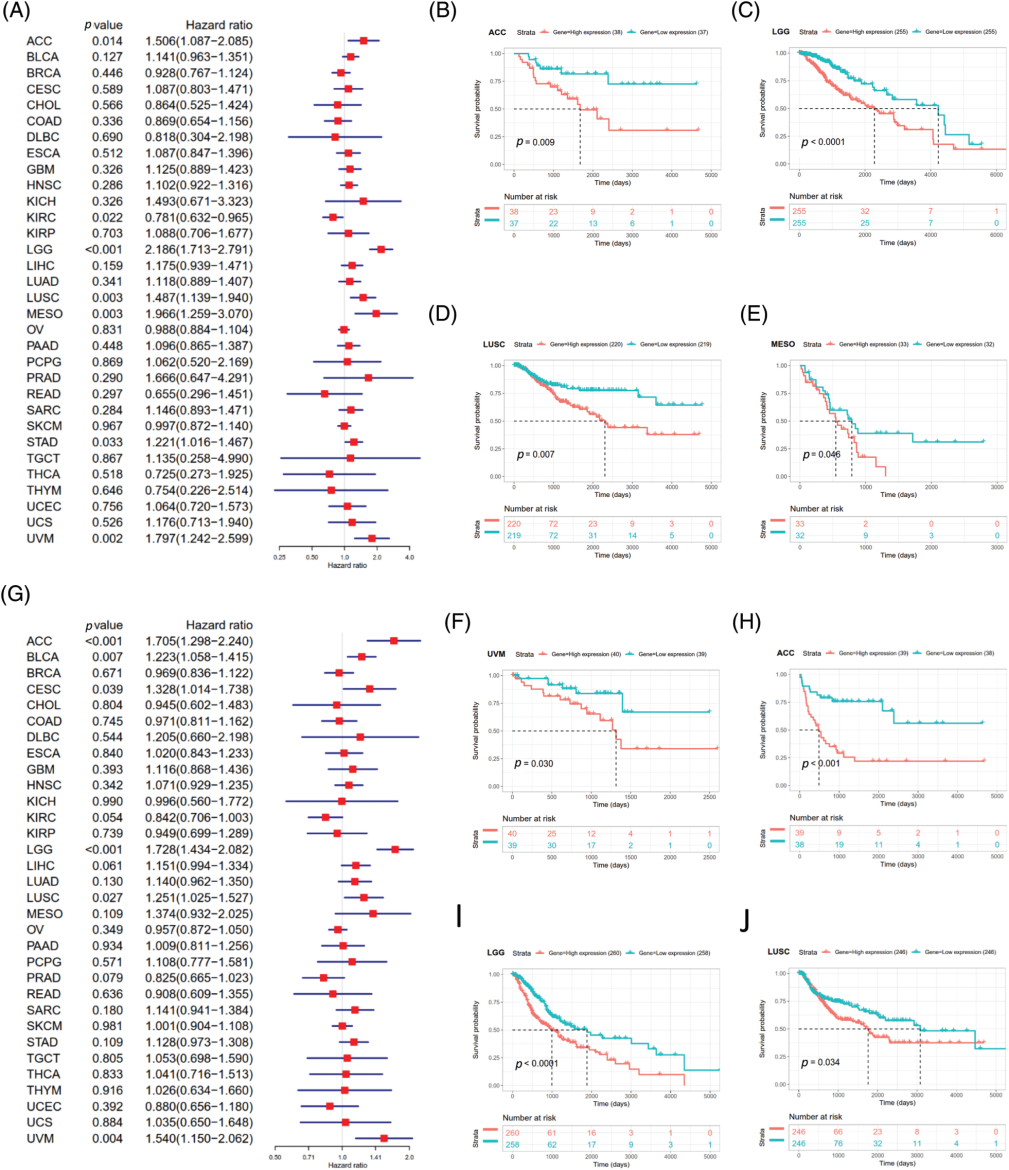
Association between SOAT1 expression and disease specific survival (DSS) or progression-free interval (PFI)in days. (A) Forest plots of DSS associations in 33 types of tumors. (B–F) Kaplan-Meier analysis of the association between SOAT1 expression and DSS. Association between SOAT1 expression and in days. (G) Forest plots of PFI associations with SOAT1 expression in 33 types of tumors. (H–J) Kaplan-Meier analysis of the association between SOAT1 expression and PFI.

DSS data analysis revealed associations between high SOAT1 expression and a poor prognosis in ACC (*p* = 0.014), LGG (*p* < 0.001), LUSC (*p* = 0.003), MESO (*p* = 0.003), STAD (*p* = 0.033), and UVM (*p* = 0.002) (Suppl. Fig. S3A). The opposite trend was observed in KIRC (*p* = 0.022) (Suppl. Fig. S3A). The results showed significant relationships between high SOAT1 expression and poor prognoses in ACC (*p* = 0.009), LGG (*p* < 0.0001), LUSC (*p* = 0.007), MESO (*p* = 0.046), and UVM (*p* = 0.030) (Suppl. Figs. S3B–S3F). Regarding DFI, enhanced SOAT1 expression associated with a poor prognosis was only observed in ACC (*p* = 0.028) and CESC (*p* = 0.036); no significant correlation was observed in any other types of cancer in the Kaplan–Meier analysis. Regarding the association between SOAT1 and PFI, forest plots showed that enhanced SOAT1 expression was related to poor PFI outcomes in ACC (*p* < 0.001), BLCA (*p* = 0.007), CESC (*p* = 0.039), LGG (*p* < 0.001), LUSC (*p* =0.027), and UVM (*p* = 0.004) (Suppl. Fig. S3G). The Kaplan–Meier analysis suggested that in ACC (*p* < 0.001), LGG (*p* < 0.0001), and LUSC (*p* = 0.034), patients with enhanced SOAT1 levels had extended survival times (Suppl. Figs. S3H–S3J). These data indicate that SOAT1 expression could be considered a biomarker for evaluating survival and prognosis in pan-cancer.

## Discussion

In this study, we showed that SOAT1 expression is greatly increased in 21 types of cancer. The results for liver cancer, lung cancer, glioblastoma, gastric cancer, and prostate cancer were similar to those obtained in previous studies [7,23–26]. Interestingly, lower SOAT1 levels in ACC, LUSC, and PCPG were observed in normal tissues, whereas the expression level was generally high throughout these tumors. In addition, using TCGA data, the Kaplan–Meier survival analysis revealed the poor prognostic effects of high SOAT1 gene expression in ACC, CESC, LGG, LIHC, MESO, STAD, UVM, LUSC, and LAML. In line with our results, Lacombe et al. reported that strong SOAT1 expression was an independent predictor of reduced OS and RFS times, reinforcing that SOAT1 has prognostic value in ACC [27]. Jiang et al. showed that SOAT1 expression is upregulated in liver cancer and further confirmed that elevated SOAT1 expression is an independent and significant risk factor for poor prognosis in LIHC [25]. Moreover, Mo et al. explored the role of SOAT1 in lung cancer [24]. They found that SOAT1 was highly expressed in human lung cancer cells and that it can promote the invasion of lung cancer cells. By contrast, high SOAT1 levels are related to a good prognosis in patients with KIRC [28].

The emerging view regarding cancer is that metabolic reprogramming evolves as tumors evolve from precancerous tissues to invasive cancers and metastatic tumors [29]. Many studies have demonstrated that cholesterol metabolism is regulated by oncogenes and tumor suppressors and have revealed the internal mechanism of cholesterol metabolic reprogramming in tumors [30]. Enhanced cholesterol synthesis and consumption contribute to oncogenesis and tumor progression [31]. Abnormal alterations in metabolic enzymes, usually acting as executors of energy metabolism, drive cancer progression [23]. SOAT1, a rate-limiting enzyme in the mevalonate pathway, plays a key regulatory role in these processes. The main function of SOAT1 is converting excess cholesterol into cholesterol esters and storing them in cell solute lipid droplets [32]. High expression of SOAT1 has been shown to be associated with lipid metabolic-related activities. SOAT1 also induces the expression of SREBP1 and SREBP2, which further enhance the level of VEGFC and ultimately contribute to invasion in gastric cancer [23]. Oni et al. found that downregulation of SOAT1 expression impaired pancreatic ductal adenocarcinoma cell proliferation by activating the mevalonate pathway that converts cholesterol to inert cholesterol [33]. Moreover, mitotane, an inhibitor of SOAT1, was used as an orphan drug for the treatment of ACC and suppresses steroidogenesis [34].

In addition, cholesterol is essential in immune cells for regulating inflammatory and innate immunity responses [35], and cholesterol is necessary for T cell receptor (TCR) aggregation and T cell immune synapse formation [36]. Ma et al. found that in cholesterol-rich lung cancer tissues, CD8+ T cells in the tumor tissue absorb excess cholesterol, which induces their failure [37]. SOAT1 has also been recognized as a major enzyme involved in cholesterol esterification. Inhibition of SOAT1 leads to recovered function and enhanced T cell proliferation in melanoma model mice [38]. In addition, Schmidt et al. showed that SOAT inhibition could reduce neutral lipid droplets of CD8+ T cells, promote lipid microdomains, enhance TCR signal transduction and TCR-independent bioenergetics, and play an anti-tumor role [39]. In glioma, increased SOAT1 expression correlated with multiple infiltrating immune cells [40]. Our study found that SOAT1 significantly correlated with the infiltration of T cells and other immune cells in various cancer types. In addition, enhanced SOAT1 was significantly correlated with immune checkpoint molecules in various cancers.

Tumor mutation burden (TMB) is a recently identified independent biomarker found in various tumors that can guide immunotherapy [41]. Patients with high TMB benefit more from immune-checkpoint treatment [20]. TMB reflects the total number of mutations, including substitutions, insertions, and deletions, per megabase in the exons from the genome of tumor cells. Numerous somatic mutations can generate neoantigens that promote T cell activations and elicit an immune response [42]. Then, as genetic variants accumulate, more neoantigens are generated and the immune system is more likely to recognize them. MSI is another useful biomarker in evaluating the effect of therapy targeting immune checkpoints [43]. High-frequency MSI has been demonstrated to be an independent biomarker for clinical features and prognosis in colorectal cancer [44]. In this study, we analyzed the correlations between SOAT1 and TMB or MSI. The results demonstrate a link between SOAT1 levels and TMB or MSI in SKCM and STAD. In addition, SOAT1 expression was dramatically related to MSI in UCEC, CESC, HNSC, STAD, THCA, PRAD, and DLBC. This finding needs to be further explored for confirmation.

Although we analyzed data originating from different databases, this study has some limitations. First, even though bioinformatic analyses have provided some relevant insights into SOAT1 in pan-cancer, *in vitro* or *in vivo* experiments are required to validate these findings. Second, despite that SOAT1 expression was demonstrated to relate to immune and clinical survival in pan-cancer, we are uncertain whether SOAT1 affects clinical survival through an immune pathway.

In summary, we first analyzed the correlation and prognostic significance of SOAT1 gene expression in a variety of tumors, indicating that SOAT1 could be used as a potential prognostic biomarker in various cancers and that its level will determine different outcomes; further investigation into the specific role of SOAT1 in different cancer types is necessary. Moreover, our findings shed light on the association between SOAT1 expression and TMB, MSI, or infiltrated immune cells, although its effect on tumor immunity varied. In the future, prospective studies are needed to confirm these findings and provide references for immune-based anticancer strategies.

## Supplementary Materials

SUPPLEMENTARY TABLE S1 Correlations between SOAT1 and immune cells in pan-cancer.

## Data Availability

Publicly available datasets were analyzed in this study. This data can be found here: The Cancer Genome Altas (https://portal.gdc.cancer.gov/).
